# Modulating cancer stemness provides luminal a breast cancer cells with HER2 positive-like features

**DOI:** 10.7150/jca.37117

**Published:** 2020-01-01

**Authors:** Yi Mei, Dongyan Cai, Xiaofeng Dai

**Affiliations:** 1 Wuxi School of Medicine, Jiangnan University, Wuxi, China; 2 School of Biotechnology, Jiangnan University, Wuxi, China; 3 Department of Oncology, Affiliated Hospital of Jiangnan University, Wuxi, China

**Keywords:** breast cancer, cancer stemness, progenitor cells, CRISPR/dCas9, therapeutic modality

## Abstract

Breast cancers can be classified into luminal A, luminal B, HER2 positive and triple-negative subtypes, each with a distinct therapeutic response. Tumor stemness drives cancer malignancy that challenges cancer control. Understanding the revolutionary relationships driven by tumor stemness among breast cancer subtypes is fundamental to identifying feasible therapeutic modalities for each breast cancer subtype.

Utilizing the endogenous tRNA-processing system, we established a multiplexing CRISPR/dCas9 system in breast cancer cells, and applied it to a four-gene panel controlling cell potency, i.e., *OCT4*, *KLF*, *MYC*, *SOX2*. The stable cell strain, OKMS#1 was obtained through concomitantly over-expressing these genes in luminal A breast cancer cells. OKMS#1 cells showed increased invasion, proliferation and cancer stemness, shared similar drug response pattern with HER2 positive cells, and exhibited altered MAPK and enhanced NFkB signaling. This study contributes in providing an efficient multiplexing CRISPR/dCas9 system that enriches our genetic modulation tool box, and suggests that HER2 positive cells are potential progenitors of luminal A cells and that these two breast cancer subtypes may share similar treatment strategies once rewired between the two states. Our results also implicate that triple negative breast cancer cells, though sharing similar cancer stemness with HER2 positive cells, represent a distinct type of disease and require unique treatment solutions.

## Introduction

Cancer stem cells (CSCs) are immortal cells within a tumor that can self-renew and give rise to the bulk cancer cells 1, 2. Tumors with more CSCs are phenotypically more malignant and more likely to establish resistance to many treatment modalities 3, 4. Therefore, targeting the CSC cohort or reducing cancer stemness is an attractive onco-therapeutic strategy.

Breast cancer is not a single disease but comprised of at least four subtypes, i.e., luminal A, luminal B, HER2 positive and triple negative, with distinct clinical outcomes and treatment responses 5, 6. HER2 positive and triple negative breast tumors are pathologically more malignant than the luminal subtypes, with the tumor grade of the former being typically 2 or 3 and that of the latter being 1 or 2 6. Although both HER2 positive and triple negative tumors are enriched with CSCs 5, they have distinct therapeutic responses. That is, while HER2 positive breast cancers can be effectively targeted with Trastuzumab 7 and sometimes with Tamoxifen^8^, triple negative cancers lack effective targeted treatment due to its lack of cell surface receptors such as estrogen receptor (ER), progesterone receptor (PR) and human epithelial receptor 2 (HER2). Such discrepancies between phenotypic response and molecular profiling suggest distinct revolutionary paths of both subtypes towards high stemness. Uncovering mechanisms underlying such discrepancies could considerably advance our understandings on the pathogenesis of both diseases that could ultimately lead to the effective control of both HER2 positive and triple negative subtypes. Taking luminal A cells (the least malignant subtype with known cure) as the control, we are motivated to study whether these cells can be switched to the HER2 positive or triple negative subtype if cells' stemness was increased.

*OCT4, KLF4, MYC* and *SOX2* were originally found capable of inducing iPS (induced pluripotent stem) cells 9 and have been widely applied for cell stemness reprogramming. For instance, *OCT4* overexpression in normal breast cells could empower cells with tumor-initiating and colonization capabilities 10, and *SOX2* over-expression could lead breast cancer cells to a more stem-like state 11. In compliance with our motivation in exploring subtype rewiring among breast cancers, we are interested in utilizing this four-gene panel coupled with the CRISPR technique in our study.

CRISPR technology offers us a precise genome-editing tool, and the use of the CRISPR-dCas9 system that lacks the shear activity could enable us to modulate the expression of targeted genes in a mild mode. For example, simultaneous suppression of multiple genes has been achieved using CRISPR/Cas9 and CRISPR/dCas9 in *Streptomyces* for functional gene screening and metabolic engineering editing 12. Concomitant transcriptional activation or inhibition of multiple genes has been achieved using the CRSIPR/dCas9 system in plants 13. In 2015, Xie et al. successfully demonstrated in rice that tandemly arranged tRNA-gRNA structures could be efficiently and accurately processed into sgRNAs of the desired targeting sequences *in vivo* to guide Cas9 in the editing of multiple chromosomal targets 14. An improved dCas9 system enabling simultaneous and precise* in vivo* transcriptional activation of multiple genes and long noncoding RNAs (lncRNAs) was established and applied in the nervous system 15. Despite the intensive efforts on multiplexing CRISPR system design and applications, relatively few study has reported the design and use of multiplexing CRISPR/dCas9 system in breast cancers 16. We are thus motivated to establish a multiplexing CRISPR/dCas9 system and apply it to study the phylogenetic relationship among breast cancer subtypes driven by cancer stemness.

## Materials and Methods

**Figure 1:**
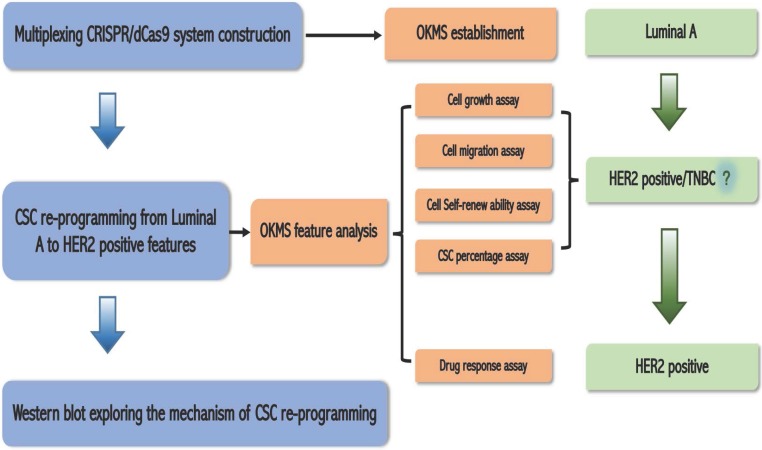
** Schematic illustration of the logic of this study.** We firstly established a multiplexing CRISPR/dCas9 system taking advantage of the endogenous tRNA-processing system; then we constructed the OKMS cell line from luminal A cells, found from cell growth, migration, cell self-renew ability, and cancer stem cell (CSC) percentage assays that OKMS cells showed HER2 positive or triple negative breast cancer (TNBC) features, and confirmed from drug response assay that OKMS cells showed HER2 positive properties; we explored the mechanism that led to cancer stem cell (CSC) rewiring using western blot.

The study design of this work is illustrated in** Figure 1**.

### Cell culture

Three human breast cancer cell lines, i.e., MCF7 (luminal A), SKBR3 (HER-2 positive) and MDAMB231 (triple negative) were used in this study, which were purchased from American Type Culture Collection (Manassas, VA, USA). MCF7 and MDAMB231 cells were maintained in Dulbecco's Modified Eagle Medium (DMEM, HyClone, American) supplemented with 10% fetal bovine serum (FBS, Lonsera, Shanghai, China) and antibiotics at 37℃ in 5% CO_2_, respectively, SKBR3 cells were maintained in Roswell Park Memorial Institute (RPMI, HyClone, American) supplemented with 10% FBS and antibiotics at 37℃ in 5% CO_2_.

### Construction of multiplexing sgRNA plasmid

The multiplexing sgRNA (small guide RNA) was constructed taking advantages of the principles of the endogenous tRNA self-shearing system. The constructed multi-gene multi-sgRNA fragment was double-cut using BbsI, resulting in two sticky ends GTGG and GTTT, following ligation with the BbsI double-cut plasmid vector pLenti-U6-sgRNA-PGK-Neo. The sgRNA sequences of *OCT4, KLF4, MYC* and *SOX2* are listed in**Table S1.**

### Cell transfection

The constructed CRISPR plasmid was diluted using 200 μl of serum-free DMEM following cell transfection using Lipofectamine^TM^ 2000 (ThermoFisher Scientific, China), with the ratio between the plasmid and Lipofectamine^TM^ 2000 being 1:3. The plasmid-reagent mixture was let stand still for 20 min to form chelates. The medium was renewed after 4 h of transfection, and supplemented with 200 mg/ml of G418 and 0.1 mg/ml of puromycin antibiotic after 24 h for screening.

### qRT-PCR

After digesting the transfected cells in a 6-well plate with trazol, the total RNA was extracted using the RNA extraction kit (TAKARA, Japan) according to the manufactor's prococol. 1 ug of the extracted RNA was used for reverse transcription to obtain cDNA using TAKARA PrimerScript RT reagent KIT (TAKARA, Japan) as per manufacturer's protocol. The cDNA was diluted to a final concentration of 10 ng/μl, and the SYBR Mix was melted at the room temperature. The PCR reaction mixture was prepared which contains 5 μl SYBR premix Ex Taq II, 0.4 μl forward and 0.4 μl reverse primers, 0.2 μl ROX reference dye and 2μl water. 8 μl PCR reaction mixture was added to the 96-well plate/eight-well strip according to the reaction layout, and 2 μl of the template cDNA solution was added. The 96-well plate or the 8-hole strip cover was sealed with a transparent film, mixed using vortex and centrifuged to ensure that the solution was concentrated at the tube bottom. The PCR reaction was run using ABI 7500 according to the thermal cycling conditions that is consisted of 2 min at 95℃, 5s at 95℃ and 30s at 60℃ for 40 cycles following 15s at 72℃. After the cycling protocol, the final step was applied to all reactions by continuously monitoring fluorescence through the dissociation temperature of the PCR product at a temperature transition rate of 0.3℃ to generate a melting curve. The qPCR primers for each gene are listed in **Table S2**.

### Western blotting

Trypsin-digested cells were centrifuged, re-suspended in pre-cooled phosphate buffered saline (PBS), supplemented with 100 μL cell lysate (containing 1% phenylmethylsulfonyl fluoride (PMSF)) in each well of a six-well plate, placed on ice and lysed for about 10 sec. The mixture was centrifuged at 10000 r·min^-1^ at 4 °C for 5 min. The supernatant was carefully absorbed, and the concentration of the protein samples was determined according to the BCA kit (ThermoFisher Scientific, China), 5× loading buffer was added to protein samples followed by 5 min boiling. The glue was loaded after cooling. The gel was cut into the appropriate size. A large PVDF film was cut out followed by methanol treatment. The treated film was immersed in the transfer buffer together with the filter paper. The membrane was transferred at a constant flow of 200 mA for 90 min, washed using Tris-buffered saline Tween-20 (TBST) for 3 times. The blocking solution was prepared by supplementing TBST solution with 5% BSA, and used to block the membrane at the room temperature for 120 min. The membrane was washed for 3 times, placed in the primary antibody diluted solution overnight, washed again for 3 times, placed it in the secondary antibody dilution solution for 120 min, and added to the ECL color development solution (Absin, China) for detection.

The secondary antibody and primary antibodies against GAPDH, OCT4, KLF4, MYC, SOX2, ER, HER2 were purchased from Proteintech, and primary antibodies targeting p65, phospho-p65, ERK1/2, phospho-ERK1/2, JNK, phospho-JNK, p38, phospho-p38 MAPK were ordered from Cell Signaling Technology.

### Cell migration assay

After cells were plated in a 6-well plate, the medium was aspirated. Cells were drawn in a straight line using a pipette tip, washed three times using PBS, and cultured in a medium supplemented with 1% serum. Photographs were taken at 0h, 8h, 16h, and 24h, respectively.

### Cell proliferation assay

Cells were plated in a 96-well plate, with the number of cells per well-being 4000. The medium was refreshed at 24h, 48h, and 72h, followed by addition of 5ul of CCK8 (GLPBIO, USA). The absorbance was measured at 450 nm after incubation at 37 °C for 2 h.

### Cell sphericity counting

Cells of the control and the experimental group were resuspended in a serum-free globular medium pre-warmed at 37 °C (concentration: 2×10^3^ cells·mL^-1^), and spread at 200 μL per well. The 96-well ultra-low adhesion plate was cultured for 7 days in a CO_2_ incubator, and photographed using the inverted phase contrast microscope (Olympus, Germany) to calculate cell sphericity.

### Detection of stem cell percentage

Cells were prepared in 6-well plates with 3×10^5^ cells allocated in each well. The plate was placed in a CO_2_ incubator for 24 h. After aspirating the medium, cells were washed twice with PBS, followed by digestion with trypsin (excluding ethylenediaminetetraacetic acid (EDTA)). Cells were centrifuged at 1000 r·min^-1^ for 5 min, the supernatant was discarded and cells were resuspended in PBS. Cells were centrifuged at 1000 r·min^-1^ for 5 min, the supernatant was discarded and cells were resuspended in the assay buffer of the ALDEFLUOR kit (STEMCELL, Canada) to prepare a cell suspension of 5×10^5^ cells·mL^-1^. 500 μL of suspension was used as one cell sample and added to each of two tubes. 10 μL of diethylaminobenzaldehyde (DEAB) was added in one tube, 2.5 μL of activated fluoroboron dipyrrole-aminoacetaldehyde-acetic acid diethyl amino malonate (BAAA) was added to both tubes. Both tubes were incubated at 37 °C for 30-40 min in the dark, centrifuged at 1000 r·min^-1^ for 5 min. The supernatant was discarded and cells were resuspended in 500 μL of assay buffer for each tube to prepare one cell sample. The flow tube was preserved under hypothermic condition, and CSC proportion was measured using a flow cytometer.

## Results

### Multiplexing CRISPR/dCas9 system construction

**Figure 2:**
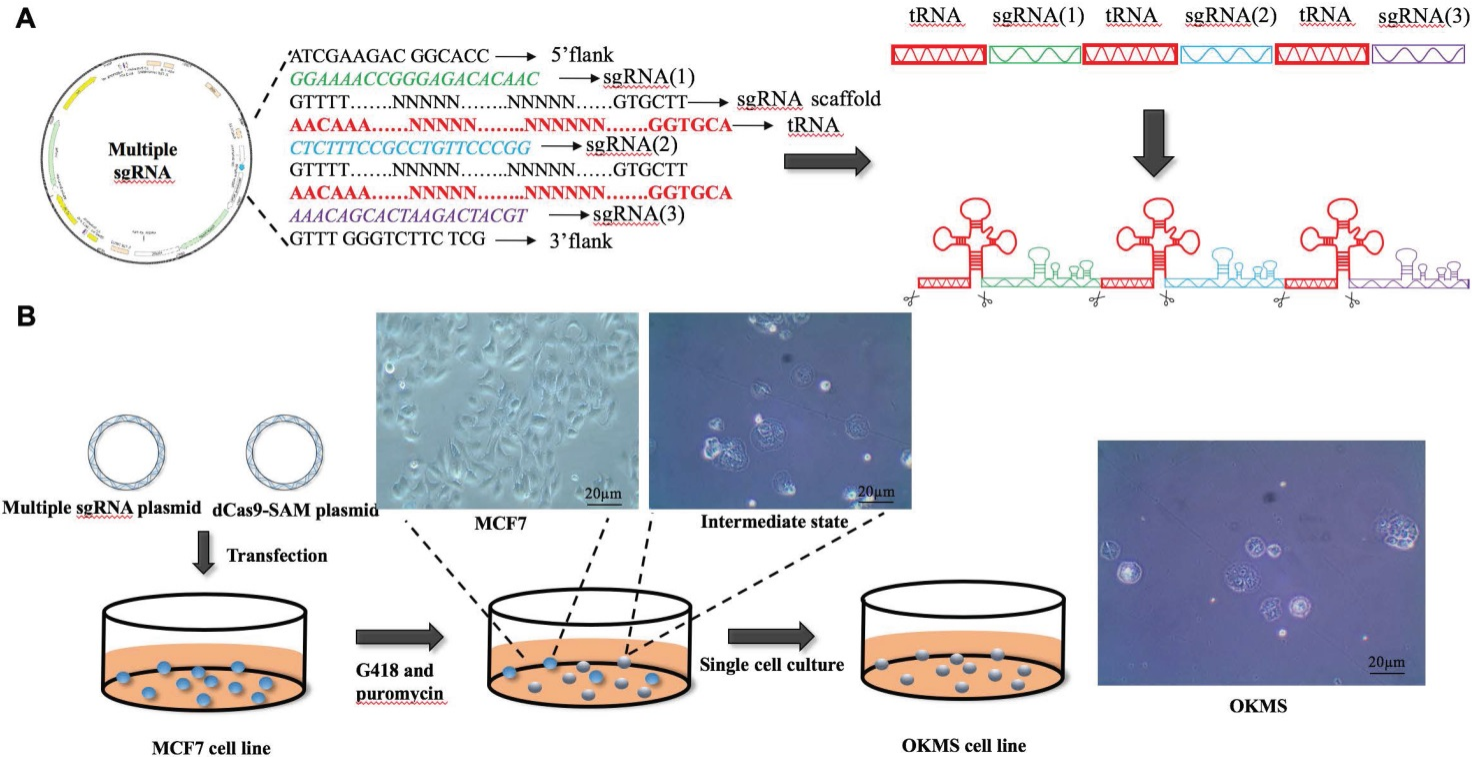
** The plasmid design and experimental procedure in the establishment of OKMS cells.** A) Design of the multi-sgRNA plasmid. B) Transfection and selection process in the establishment of stable OKMS cells.

**Figure 3:**
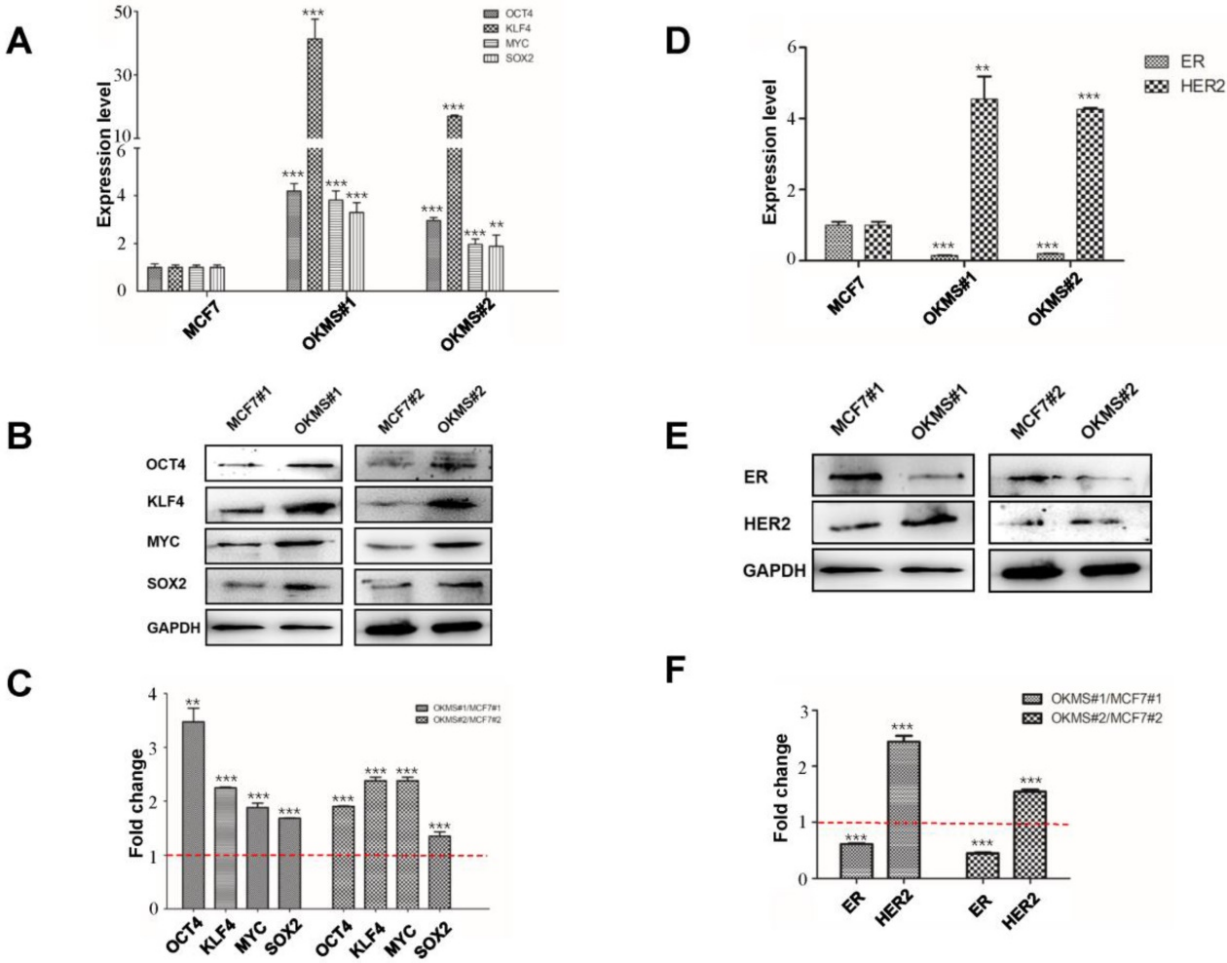
** Expression of cell stemness and subtyping markers in stable OKMS cells.** A) Expression of cell stemness markers (OCT4, KLF4, MYC and SOX2) at the A) transcriptional and B) translational levels. Expression of subtyping markers (ER, HER2) at the D) transcriptional and E) translational levels. Western blot signalling intensities normalized by that of GAPDH in OKMS cells are plotted in C) for stemness markers (OCT4, KLF4, MYC and SOX2) and in F) for subtyping markers (ER, HER2). Bars represent mean ± SD of fold change from at least three independent experiments, and ** (0.01 <P < 0.05) and *** (P<0.01) represent student t-test p values computed using raw ratios.

To concomitantly regulate the expression of the four gene panel, i.e., *OCT4, KLF4, MYC, SOX2*, we established a simple yet robust platform to boost the CRISPR/dCas9 multiplex editing capability taking advantages of the endogenous tRNA-processing system. The endogenous tRNA-processing system precisely cleaves both ends of the tRNA precursor. We engineered a multiplexing sgRNA plasmid with the four genes tandemly arrayed in a tRNA-sgRNA architecture, and transfected it together with the dCas9-SAM (synergistic activation mediator) plasmid (which functions as an engine for activating endogenous gene expression) into the breast cancer cell line MCF7. Through antibiotic screening and single cell culturing, we obtained two stable cell strains with both plasmids successfully transfected (**Figure 2B**). The results showed that the multiplexing sgRNA plasmid was efficiently and precisely processed into sgRNAs with desired 5' targeting sequences in cells, which directed dCas9 to edit multiple targets simultaneously (**Figure 2A**). Both qPCR and western blotting revealed concomitant increase in the expression of all four genes in both cell strains (**Figures 3A, 3B**), demonstrating our success in constructing the multi-gene CRISPR/dCas9 system.

### Establishment of OKMS cells

A stable cell strain concomitantly over-expressing *OCT4, KLF4, MYC, SOX2*, namely OKMS, was successfully constructed using the multi-gene CRISPR/dCas9 system. The expression of all genes in the cell strain OKMS#1 was more than three times higher than that of the control (p<0.01), among which KLF4 expression increased over 30 times. The expression of all genes in cells OKMS#2 was enhanced over double-folds as compared with the control (p<0.05) (**Figure 3A**). The same results were shown at the protein level (**Figure 3B**). The expressions of canonical breast cancer subtyping markers, ER and HER2, were significantly altered. ER was significantly down-regulated (p<0.05) and HER2 was considerably up-regulated (p < 0.01) at the transcriptional and translational levels, suggesting a switch from the luminal A-like to the HER2 positive-like phenotype. OKMS#1 was selected in the following experiments.

### Cell growth and migration behavior of OKMS cells

**Figure 4:**
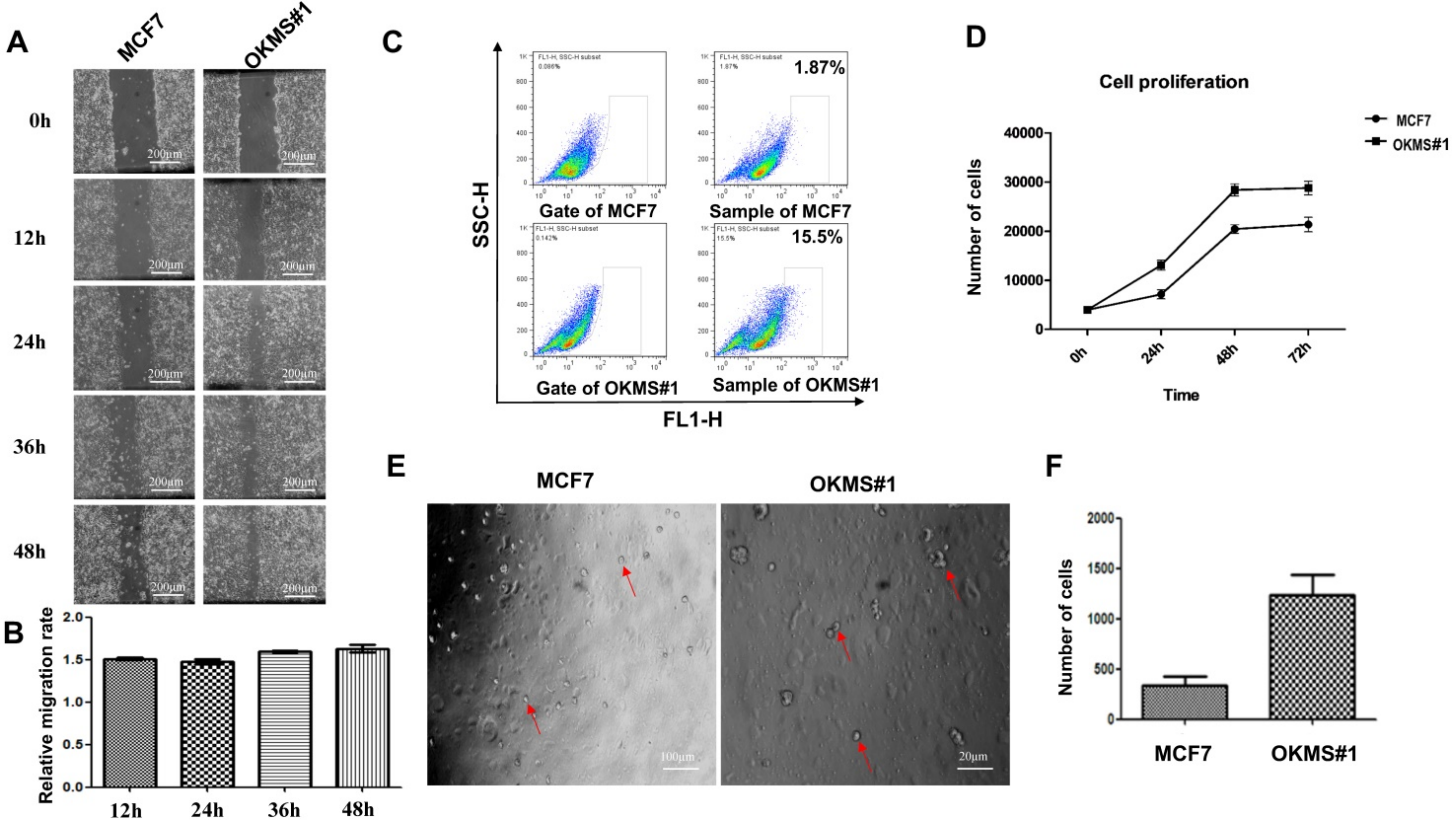
** Morphological alterations in stable OKMS cells as compared with MCF7 cells.** A) Images measuring cell migration abilities. B) Digital statistics quantitizing cell migration abilities. C) Flow analysis graph showing cell cancer stem cell percentages. D) Growth curves showing cell proliferative abilities. E) Images measuring cell self-renew abilities. F) Digital statistics quantitiziing cell self-renew abilities. Bars represent mean ± SD from at least three independent experiments.

The OKMS#1 cell line underwent a dramatic change in the morphology, i.e., from polygonal to spherical in shape, as compared with the control (**Figure 2**). Both the growth and migration abilities of OKMS#1 cells were increased up to 1.5 times than that of the control with statistical significance (p < 0.01,** Figures 4A, 4B, 4D**), where the migration was measured at 12, 24, 36 and 48 hours. The percentage of breast CSCs increased from 1.87% in MCF7 to 15.5% in OKMS#1 cells (**Figure 4C, Figure S1**). Cell sphericity increased over 2 times in OKMS#1 cells than MCF7 where they were originated from (**Figures 4E, 4F**). These results suggested that OKMS#1 cells were more malignant than MCF7 cells.

### Drug response of OKMS cells

**Figure 5:**
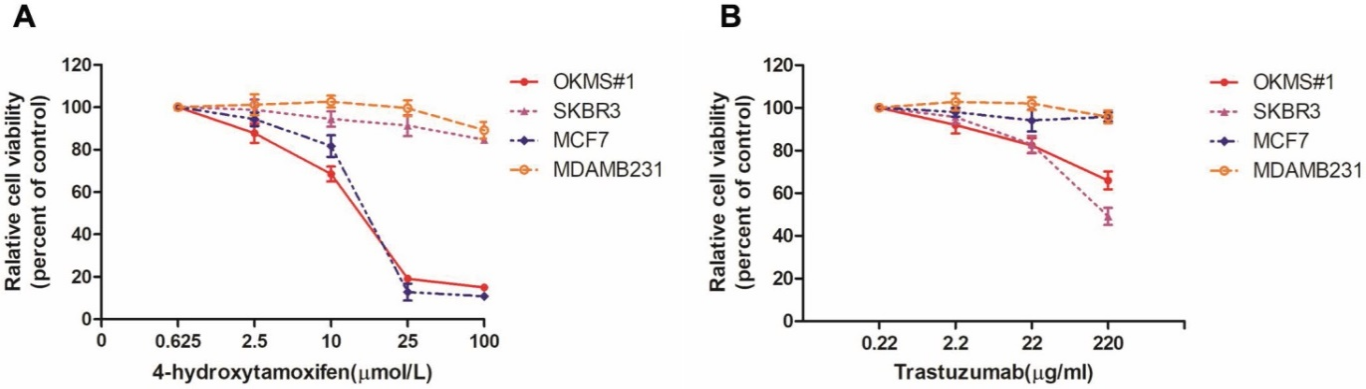
** Drug dose curves of cells in response to Tamoxifen and Trastuzumab.** Drug response curves under the treatment of A) Tamoxifen, B) Trastuzumab. MCF7, SKBR3, MDAMB231 are luminal, HER2 positive and triple negative cells, respectively, and OKMS is a stable cell line established from MCF7 by over-expressing the four-gene panel controlling cell stemness (OCT4, KLF4, MYC and SOX2).

We tested the drug sensitivity of OKMS#1, MCF7, SKBR3, MDAMB231 to two drugs canonically used for breast cancer treatment, i.e., 4-Hydrotamoxifen (a hormonal therapy) and Trastuzumab (targeted therapy against HER2 positive cells). OKMS#1, MCF7 (luminal A) and SKBR3 (HER2 positive) cells shared similar dose response curves and were sensitive to Tamoxifen after 3 days of treatment (**Figure 5A**). OKMS#1 and SKBR3 responded to Trastuzumab after 3 days of drug administration (**Figure 5B**). These results revealed that OKMS#1 cells shared similar drug response pattern with the HER2 positive cell line SKBR3.

### Reprogrammed cancer signalings in OKMS cells

**Figure 6:**
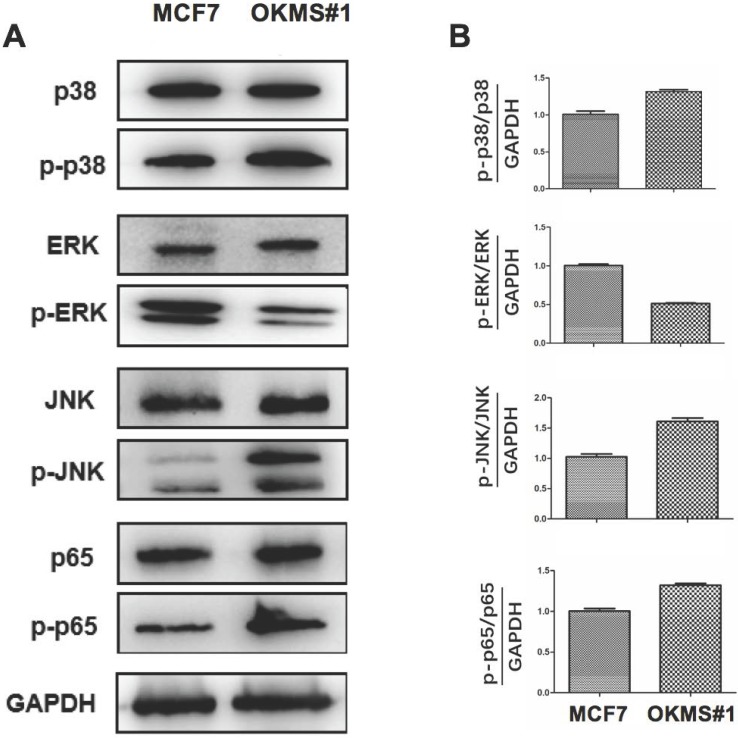
** Western blot of key signaling molecules in MAPK and NFkB pathways in OKMS cells with MCF7 cells as the control.** A) ERK, JNK, p38 are the key signaling molecules of three important MAPK pathways, and p65 is NFkB. B) Western blot signalling intensities normalized by that of GAPDH in OKMS cells.

OKMS#1 cells showed decreased ERK phosphoration (around half that of the control), increased JNK phosphoration (over 1.5 folds of the control), and slightly increased p38 and p65 phosphoration (both are around 1.3 folds of the control), suggesting that NFκB, MAPK/JNK, and MAPK/p38 were activated in OKMS#1 and MAPK/ERK was suppressed during the four-gene mediated reprogramming (**Figure 6**).

## Discussion

### HER2 positive cells are potential progenitor cells of luminal A cells

OKMS#1 cells exhibited increased proliferation (**Figure 4D**), enhanced migration (**Figures 4A, 4B**), exemplified CSC percentage and self-renew abilities (**Figures 4C, 4E, 4F**). These phenotypic changes reflect a phenotype transition from luminal A (MCF7, where OKMS#1 was derived from) to a more malignant state that is similar to HER2 positive or triple negative breast cancer cells. Drug response curves showed that OKMS#1 shared a similar pattern with the HER2 positive cell line SKBR3 (**Figure 6**) but not triple negative cells. Thus, HER2 positive cells may be potential progenitor cells of luminal A cells, and could be controlled, in principle, using the same therapeutic strategies as the luminal A subtype and eliminated if coupled with appropriate approach rewiring cancer cell stemness.

### Triple negative cells form a biologically distinct cell cohort among breast cancer cells and require distinct therapeutic strategies

As aforementioned, HER2 positive cells can ideally be switched to a less stem-like state to achieve desired outcome with hormonal therapies. Tumor subtype state transition has previously been implicated in melanoma through altering Stat3 expression 17. However, such a strategy may not be feasible to treat triple negative breast cancers, which is phenotypically distinct from the other subtypes that have efficient therapies. This imposes further challenges to the appropriate control of triple negative breast cancers that still lacks safe yet effective treatment approaches 18. Though being similar on patient survival, HER2 positive and triple negative cancers are distinct in how their CSCs are originated from, which must be reflected in their molecular profiles. Thus, comprehensive decomposition of the molecular features of triple negative cancers and comparison with that of HER2 positive cancers may lead to the identification of the unique path to the malignancy of triple negative breast cancer cells as compared with the other subtypes and, ultimately, the identification of novel approaches for the effective control of such cancers.

## Significance

**Figure 7:**
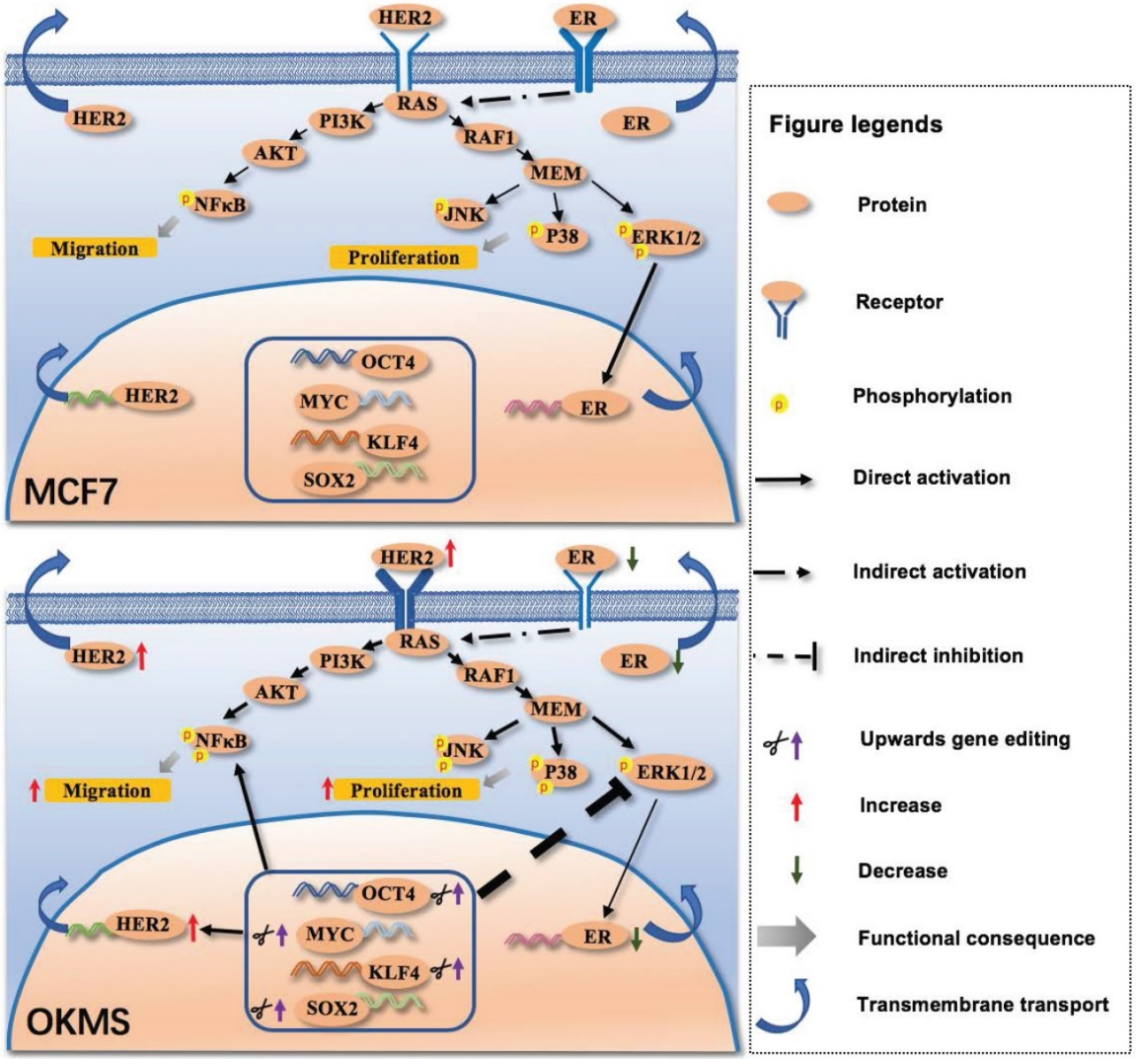
** Signaling network mediated by the four-gene panel controlling cell stemness in the transition of cancer cell states.** The four-gene panel controlling cell stemness rewires cells from a luminal A-like feature to a HER2 positive-like feature through collective alterations in MAPK/ERK, MAPK/JNK, MAPK/p38 and NFkB signalings. Once the four-gene panel was activated, HER2 expression was enhanced, leading to increased phosphorylation of NFkB, JNK and p38, and decreased ERK. Elevated NFkB signaling, and MAPK/JNK and MAPK/p38 signalings result in enhanced cell migration and proliferation. Decreased ERK lead to decreased ER expression. Therefore, by concomitantly up-regulating the four-gene panel, HER2 is over-expressed, and ER is suppressed, leading to a state change from exhibiting luminal A like features to HER2 positive like features (namely OKMS cells). Phosphorylation status is shown only for molecules examined in our experiments. The thickness of each line or arrow represents the strength of the regulatory effect, with a more thick line or arrow representing a stronger effect. The thickness of each receptor represents the expression status of each receptor, with a more thick symbol representing a more represented status.

We have successfully designed an efficient multiplexing CRISPR/dCas9 system and applied it to study the phylogenetic relationships among breast cancer subtypes as stratified by cancer stemness. Concomitant up-regulation of *OCT4, KLF4, MYC* and *SOX2* provides luminal A cells with HER2 positive-like features as observed both at the molecular profile and cellular behavior levels. This implicates that cell stemness controls the phenotypic transition between luminal A and HER2 positive breast cancer cells (**Figure 7**); and triple negative cells, though sharing similar cancer stemness with the HER2 positive subtype, represent a revolutionarily distinct disease from the other breast cancer subtypes that requires distinct therapeutic strategies for effective control.

## Supplementary Material

Supplementary tables.
