# Supplementary material for: Modulating cancer stemness provides luminal a breast cancer cells with HER2 positive-like features
Source: J Cancer. 2020 Jan 1;11(5):1162–9. doi: 10.7150/jca.37117 (PMC6959057; doi:10.7150/jca.37117)
Supplement: Supplementary file 1 — Supplementary tables. [file jcav11p1162s1.pdf]

Table S1. The sgRNA design for the modulation of *OCT4, KLF4, MYC, SOX2* in the experiments.

Type	Function	Gene	Sequence
sgRNA1	enhance	OCT4	GGAAAACCGGGAGACACAAC
sgRNA2	enhance	OCT4	GGATGTTTGCCTAATGGTGG
sgRNA1	enhance	KLF4	CTCTTTCCGCCTGTTCCCGG
sgRNA2	enhance	KLF4	CAGTTCACGCTGCACAGTGC
sgRNA1	enhance	MYC	AGCTAGAGTGCTCGGCTGCC
sgRNA2	enhance	MYC	GAACCCGGGAGGGGCGCTTA
sgRNA1	enhance	SOX2	AAACAGCACTAAGACTACGT
sgRNA2	enhance	SOX2	GCCCCCTTTCATGCAAAACC

Table S2. Primers used in qRT-PCR.

Gene	Forward primer	Reverse primer
OCT4	GGGAGATTGATAACTGGTGTGTT	GTGTATATCCCAGGGTGATCCTC
KLF4	CGGACATCAACGACGTGAG	GACGCCTTCAGCACGAACT
SOX2	GCTTAGCCTCGTCGATGAAC	AACCCCAAGATGCACAACTC
MYC	GGCTCCTGGCAAAAGGTCA	CTGCGTAGTTGTGCTGATGT
ER	CAGGCATTCGGTTTGATGAGT	TTGGACGAAGTACAGTTCCCG
HER2	TGTGACTGCCTGTCCCTACAA	CCAGACCATAGCACACTCGG
GAPDH	CCCACTCCTCCACCTTTGAC	ATGAGGTCCACCACCCTGTT
